# Update from the 5th Edition of the World Health Organization Classification of Head and Neck Tumors: Hypopharynx, Larynx, Trachea and Parapharyngeal Space

**DOI:** 10.1007/s12105-021-01405-6

**Published:** 2022-03-21

**Authors:** Nina Zidar, Nina Gale

**Affiliations:** Institute of Pathology, Faculty of Medicine, University of Ljubljana, Korytkova 2, 1000 Ljubljana, Slovenia

**Keywords:** Precancerosis, Tumors, Hypopharynx, Larynx, Trachea, WHO

## Abstract

In this article, we review the chapter on tumors of the larynx, hypopharynx, trachea and parapharyngeal space in the new edition of the WHO book, focusing on the new developments in comparison to the previous edition. Squamous cell carcinoma (SCC) and its variants are by far the most common malignancies at these locations, with very limited new insights. The most important is the introduction of new targeted treatment—checkpoint inhibitors, with a new task for pathologists, who may help to predict the response to treatment by analyzing the expression of targeted proteins in biopsy samples. Precancerous lesions remain a controversial topic and, similarly to other organs, it is acceptable to use the terms “dysplasia” or “squamous intraepithelial lesion” (SIL), but there is a slight difference between low-grade dysplasia and low-grade SIL: in the former, mild atypia must be present, while the latter also includes hyperplastic epithelium without atypia. Two approaches have been proposed: a two-tiered system with low- and high-grade dysplasia/SIL and a three-tiered system with an additional category, carcinoma in situ. We are still searching for reliable diagnostic markers to surpass the subjectivity in biopsy diagnosis, with a few potential candidate markers on the horizon, e.g., stem cell markers. Other tumors are rare at these locations, e.g., hematolymphoid, neuroendocrine and salivary gland neoplasms, and are no longer included in Chapter 3. They must be diagnosed according to criteria described in specific chapters. The same holds true for soft tissue tumors, with the exception of cartilaginous neoplasms, which are still included in Chapter 3.

## Introduction

Significant progress has been made in head and neck pathology, mostly based on new discoveries of the genetic background of tumors and other diseases, resulting in new entities and more precisely defined diagnostic, prognostic and predictive factors. Larynx, hypopharynx and trachea seem to be an exception. Despite their complex anatomic and histologic structure, tumors at these locations are usually of squamous origin and the vast majority of malignant tumors belong to squamous cell carcinoma (SCC); little if any progress has been achieved in laryngeal and hypopharyngeal SCC.

Consistently, the new edition of the WHO presents few changes in this field of head and neck pathology. Moreover, tumors other than SCC that can arise in the larynx, hypopharynx and trachea, e.g., hematolymphoid neoplasms, neuroendocrine and salivary type tumors, have been moved to specific chapters. The same holds true for soft tissue tumors, with the exception of cartilaginous neoplasms, which are still dealt with in Chapter 3.

The most important seems to be the introduction of immunomodulatory drugs in the treatment of advanced SCC of the larynx and hypopharynx and other head and neck SCC. This is of particular importance for pathologists now being asked to analyze the expression of target proteins, e.g., PD-1 and PD-L1 in biopsy material, to predict the response to targeted treatment.

In this article, we will briefly review the chapter on tumors of the larynx, hypopharynx, trachea and parapharyngeal space in the new edition of the WHO book.

## Precancerous Lesions

Traditional light microscopy remains the mainstay among prognostic factors to predict the biological behavior of precursor lesions and to guide clinicians in selecting the most appropriate treatment [1]. Recently, these lesions have been commonly designated dysplasia or squamous intraepithelial lesions (SILs) [1–6], replacing the previous terminology, e.g., squamous intraepithelial neoplasia (SIN) and laryngeal intraepithelial neoplasia (LIN) [7].

Smoking and alcohol abuse with their multiplicative combined effect, are the major risk factors [6, 8] while human papillomavirus (HPV) infection probably does not contribute significantly [9]. HPV-DNA detection is usually related only to transition HPV infection of laryngeal lesions and has no important impact in carcinogenesis [10].

Two approaches for classifying precancerous lesions have been proposed: a two-tiered system with low-grade (LG) dysplasia/SILs and high-grade (HG) dysplasia/SILs and a three-tiered system with an additional category, carcinoma in situ (CIS). LG/SILs dysplasia is characterized by augmentation of the basal and parabasal cells occupying up to the lower half of the epithelium, with minimum cellular atypias and retained spinous layer in the upper half of the epithelium. Though the terms “dysplasia” and “SILs” are generally regarded as synonymous, there is a slight difference between LG dysplasia and LG SILs: in the former, mild atypia must be present, while the latter also includes hyperplastic epithelium (with increased spinous or basal/parabasal cells) without atypia. It is, however, extremely difficult to define morphologically mild atypia on the basis of morphology alone and to distinguish LG dysplasia from LG SILS with basal/parabasal cell hyperplasia [4, 7].

HG dysplasia/SILs is characterized by variable degrees of disordered stratification and polarity of cells occupying more than half of the epithelial thickness, with variation of cellular and nuclear atypias and an increased number of mitoses within the whole epithelium. It can be divided into basaloid type with no maturation and large (spinous) type with maturation, with or without keratinization [3, 11–13].

**Fig. 1:**
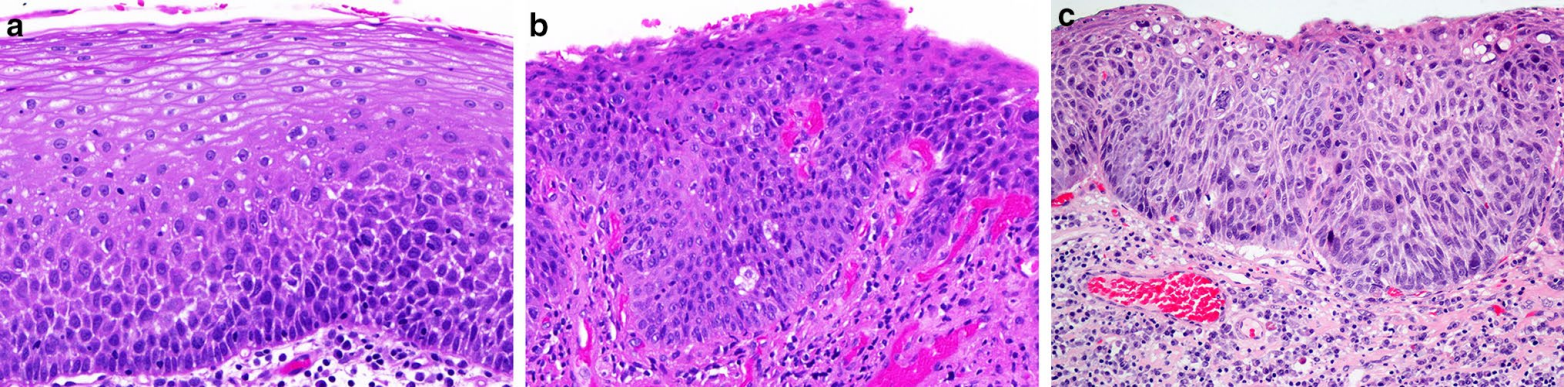
**a** Low grade dysplasia: hyperplastic epithelium with increased basal type cells, with very mild atypia, occupying lower half of epithelium. **b** High grade dysplasia: hyperplastic epithelium with increased atypical basal type cells, occupying almost entire thickness of epithelium, with preserved polarity. **c** Carcinoma in situ: parakeratotic, hyperplastic epithelium, with pronounced architectural and cellular abnormalities, with severe nuclear and cellular atypias, occupying the entire epithelial thickness

Using a two-tiered system, the category of HG dysplasia is wide, encompassing previous categories of moderate and severe dysplasia, as well as CIS [11]. This disadvantage can be resolved by introducing an additional category—CIS, and the two-tiered classification is consecutively transformed into a three-tiered classification [11, 12]. CIS is characterized by marked architectural disorder of the epithelium, conspicuous cellular atypias, increased mitotic activity with atypical mitoses, and preserved basement membrane. CIS, as an additional category, allows a pathologist to provide a diagnosis of malignancy rather than dysplasia and it is beneficial for clinicians to use radiotherapy for these most severe lesions, especially if located at specific sites (anterior commissure) and in patients with additional high risk factors [6, 13] (Fig. 1).

**Fig. 2:**
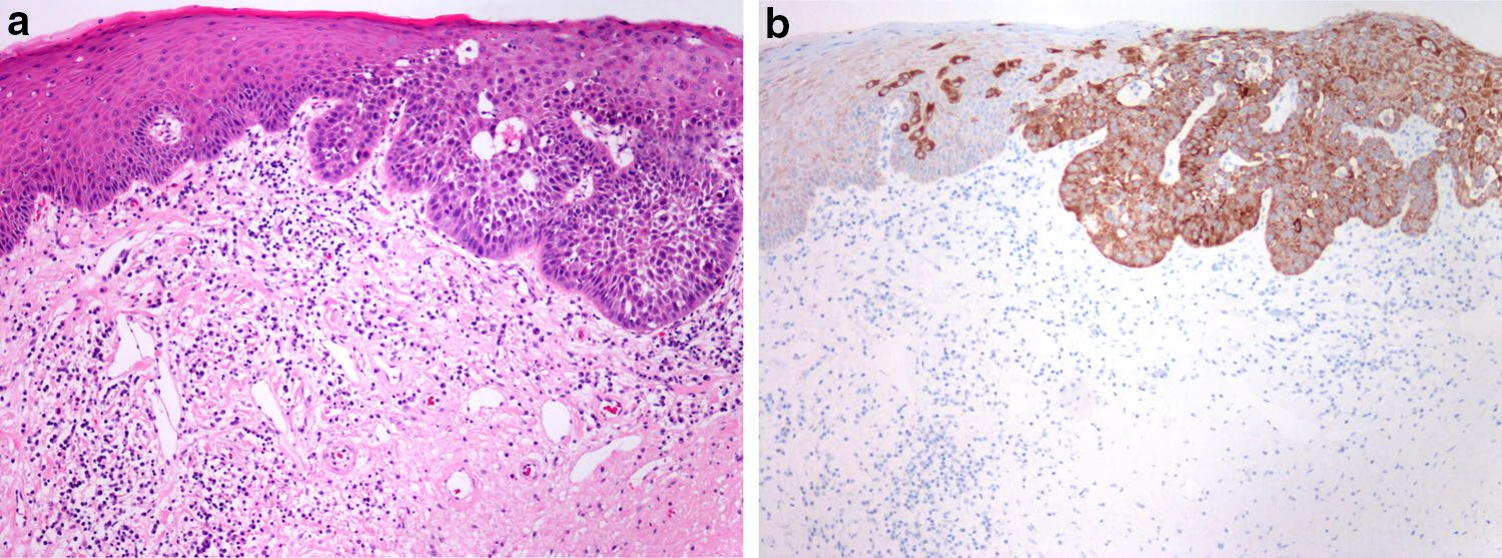
**a** Laryngeal dysplasia: transition from low-grade to high-grade dysplasia. **b** NANOG immunohistochemistry: weak expression in low-grade dysplasia, and strong expression in high-grade dysplasia

Reliable diagnostic and prognostic markers for grading precancerous lesions are still lacking, but recent studies presented some promising new candidates, e.g., melanoma-associated antigens A (MAGE-A) family and NANOG. MAGE-A expression by immunohistochemistry and RT-PCR was associated with the risk of malignant transformation in laryngeal and oral leukoplakia [14]. NANOG is a stem cell marker, showing no immunohistochemical staining of the normal mucosa, weak staining in LG dysplasia, and strong staining in HG dysplasia (Fig. 2) [15, 16]. Strong NANOG expression showed association with laryngeal cancer risk superior to the histological classification [16].

**Fig. 3:**
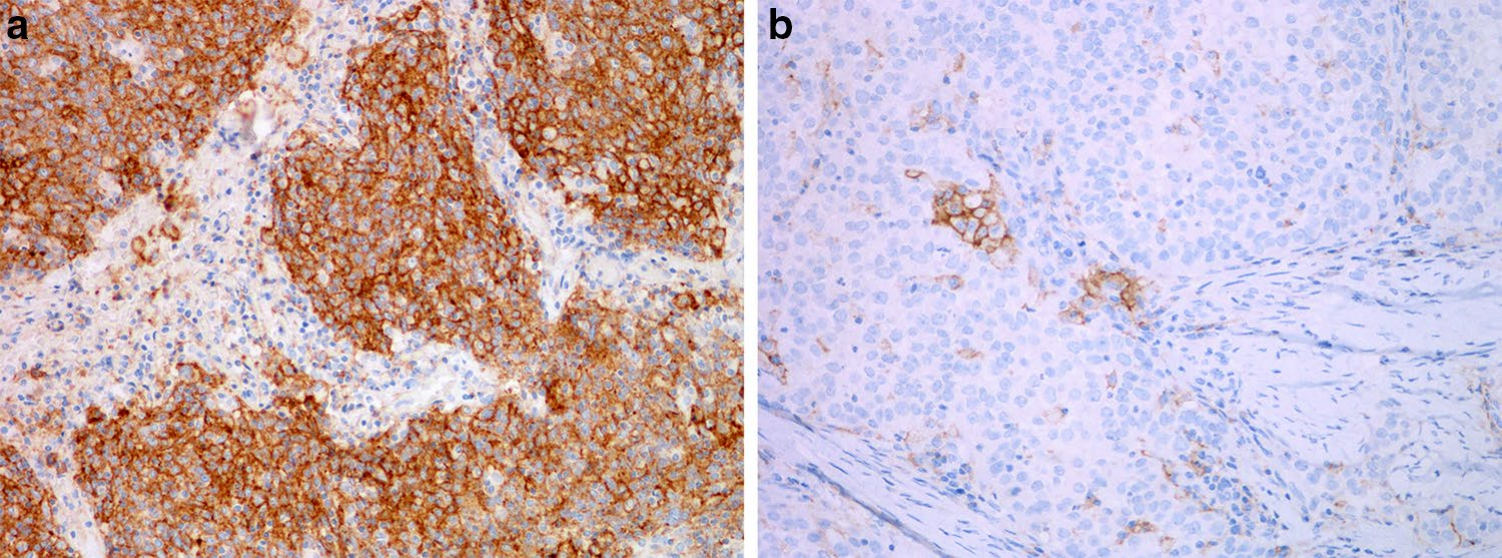
PD-L1 immunohistochemistry in squamous cell carcinoma of the larynx. **a** Strong diffuse expression in tumor cells and in some immune cells. **b** Focal expression in tumor cells and in some immune cells

Data of dysplasia progression to SCC vary in different studies. The histological grade of dysplasia, which is not always classified uniformly, the effects of different treatment modalities, as well as effects of major risk factors need to be considered in assessing risk and interval of malignant transformation [17]. It is therefore not surprising that the rate of malignant transformation in 7 studies varies significantly: in mild dysplasia from 0 to 41.7%, in moderate dysplasia from 0 to 48%, in severe dysplasia from 14.3 to 44.4%, and in CIS from 11.1 to 75% [18]. The largest published retrospective study demonstrated a highly significant difference in the risk of malignant progression between LG dysplasia and HG dysplasia, ranging from 1.6 to 12.5%. Patients with CIS were not included in the study because they were treated differently than patients with HG- or LG dysplasia [2] (Fig. 3).

## Conventional Squamous Cell Carcinoma

SCC remains the most common tumor of the larynx, hypopharynx and trachea, being the second most common respiratory tract cancer. There has been a slight decrease in the incidence of laryngeal SCC. Etiologically, these cancers are strongly associated with cigarette smoking and alcohol intake. Other factors play a limited role in the development of SCC at these locations, including gastroesophageal reflux, HPV and EBV. Microscopical assessment of these tumors has not changed for a long time. We still grade them according to modified Broder’s criteria into well, moderately and poorly differentiated SCC, with limited prognostic significance. Lymphatic, perineural, vascular invasion and lymphatic tumor infiltration are considered important prognostic factors, as well as extranodal extension in lymph node metastases [19].

Unless poorly differentiated, the diagnosis is usually straightforward. If poorly differentiated, neuroendocrine carcinoma, melanoma and lymphoma must be excluded using immunohistochemistry. Another differential possibility is NUT carcinoma, particularly when an abrupt transition between undifferentiated and squamous areas is present [20].

SCCs of the larynx, hypopharynx and trachea are often diagnosed late and are therefore associated with substantial morbidity and mortality which have not changed significantly in recent decades. The available treatment modalities traditionally include surgery, radiation and systemic therapy, mainly consisted of platinum-based chemotherapy, taxanes and anti-EGFR antibody cetuximab [21]. Recently, immunotherapy has emerged aiming to restore the ability of the immune system to identify and destroy tumor cells [22]. Particular attention has been given to antigens associated with cytotoxic T lymphocytes-4 (CTLR-4), programmed cell death ligand 1 (PD-L1), indoleamin-2.3-dioxygenase (IDO), and some others as potential targets for treatment [23]. They can modulate the immune response to cancer and belong to “immune checkpoints”, representing a basis for new therapies [23].

The anti PD-1 antibodies, nivolumab and pembrolizumab, are the first immune checkpoint inhibitors approved for the treatment of patients with recurrent and/or unresectable metastatic head and neck SCC [24, 25]. The recommendation for the first line therapy (without surgery or radiotherapy option) is based on the results of the first-line KEYNOTE-048 study, which included 882 patients with untreated recurrent or metastatic SCC of the head and neck, among them 237 and 157 patients with SCC of the larynx and hypopharynx, respectively. It showed that pembrolizumab monotherapy or combined with platinum and 5-fluorouracil is an appropriate first-line treatment for recurrent or metastatic head and neck SCC, and pembrolizumab monotherapy is an appropriate first-line treatment for PD-L1-positive recurrent or metastatic head and neck SCC [26].

Not all patients respond well to treatment with nivolumab and pembrolizumab. One of the factors that may help to predict the response to treatment is the expression of PD-L1 on tumor and immune cells. It is currently advised to calculate a combined positive score (CPS) on the basis of immunohistochemistry using antibody 22C3. CPS is defined as the number of PD-L1-positive cells (tumor cells, lymphocytes, and macrophages) divided by the total number of tumor cells × 100. With a well-defined approach, it is easier to compare success rates of therapy. Previous studies have shown a marked variation in the percentage of PD-L1 positive cases of head and neck SCC with different antibodies, ranging from 0 to 25.1%, even within the same tumor category, and the KEYNOTE-048 study suggests that CPS is more specific than the tumor proportional score (TPS) [26, 27].

Recent trials have demonstrated that PD-L1 expression is associated with an increased objective response rates in patients with CPS ≥ 1, with a better response with CPS ≥ 20 [26, 27]. However, the lack of response in some PD-L1 positive patients clearly indicates that other factors are involved in the resistence to treatment with check-point inhibitors. Apart from the expression of PD-L1 in tumor cells, gender and HPV status have been reported to play a role in treatment response, being more efficient in female patients and in HPV-positive tumors [28].

It is expected that in the future, pathologists may play a crucial role in analyzing biopsies in order to predict response to targeted treatment and help to identify poor and good responders to a specific treatment [25].

## Subtypes of Squamous Cell Carcinoma

The subtypes of SCC include verrucous carcinoma, basaloid SCC, papillary SCC, spindle cell squamous carcinoma, adenosquamous carcinoma, and lymphoepithelial carcinoma [30–35]. In the larynx, hypopharynx and trachea, they are mostly related to smoking and alcohol abuse, and only exceptionally to infection with HPV or EBV. SCC subtypes are true clinico-pathologic entities, with a prognostic significance and well-defined differential diagnosis. Many tumors that must be considered in differential diagnosis of SCC subtypes have now a well-defined genetic background (e.g., salivary gland tumors, sarcomas, NUT carcinoma), which might be helpful in difficult cases.

*Verrucous carcinoma* is a variant of well-differentiated SCC that lacks cytologic features of malignancy and does not metastasize. It is characterized by a lateral spread and invasion below the level of adjacent epithelium. It is now clear that, regardless of location, it is not associated with infection with HPV [36, 37]. The diagnosis of verrucous carcinoma is extremely difficult, particularly in small biopsy specimens. The differential diagnoses include verrucous hyperplasia that lacks evidence of invasion below the level of the surrounding epithelium, and conventional SCC which shows clear atypia. Verrucous carcinoma has a significantly better prognosis than conventional SCC [38, 39].

*Basaloid squamous cell carcinoma* is an aggressive variant of SCC with prominent basaloid morphology and the presence of squamous differentiation, often with myxoid or hyaline stromal alterations. In the larynx and hypopharynx, it is not associated with HPV infection [40, 41]. It must be differentiated from neuroendocrine carcinoma and from HPV-positive nonkeratinizing SCC extending from the oropharynx, which has a better prognosis than basaloid SCC [40]. Most studies have shown a worse prognosis of basaloid SCC in comparison to conventional SCC [42–44].

*Papillary squamous cell carcinoma* is characterized by exophytic growth, composed of papillae covered by atypical stratified squamous or immature basaloid epithelium. It can be associated with high-risk HPV infection, but it is not clear whether or not the presence of HPV affects the prognosis [45–47]. The invasion, if present, can be difficult to prove, particularly in biopsy specimens, and usually consists of irregular nests of nonkeratinizing SCC. Papillary SCC must be distinguished from verrucous carcinoma, which is usually keratinizing and lacks atypia, and from papilloma, which typically does not show atypia, which is always present in papillary SCC. Because of the exophytic growth, papillary SCC is usually diagnosed at an early stage and has a better prognosis than conventional SCC [45].

*Spindle cell squamous carcinoma* is composed of spindle or pleomorphic cells, usually with a SCC component. It is now regarded as a SCC that has underdone epithelial-mesenchymal transition [48]. The diagnosis is straightforward when both spindle and SCC components are present. If not, it must be differentiated from sarcomas, which are exceedingly rare in the larynx, hypopharynx and trachea. A malignant spindle cell tumor at these locations is considered spindle cell squamous carcinoma until proven otherwise. Another differential diagnostic possibility is inflammatory myofibroblastic tumor, which is characterized by various genetic alterations, affecting *ALK, ROS1* and *NTRK3* [47], which are not present in spindle cell carcinoma. In the larynx, the prognosis for spindle cell carcinoma is comparable or slightly better than for conventional SCC. This has been attributed to the fact that spindle cell carcinoma in the larynx is often polypoid and of glottic origin. It presents earlier due to functional impairment of voice, lacks deep invasion and rarely metastasizes due to vocal cord anatomy, with poor lymphatic supply. In the hypopharynx, the prognosis is worse than for conventional SCC [50–52].

*Adenosquamous carcinoma* is a biphasic tumor showing both squamous and glandular differentiation. The SCC component is usually superficial, either as carcinoma in situ or as an invasive SCC, and the glandular component is usually located in a deeper part of the tumor. The two components occur in close proximity but are generally distinct and separate. It must be distinguished from mucoepidermoid carcinoma, which has a better prognosis. Molecular analysis might be helpful: *MAML2* translocation is characteristic of mucoepidermoid carcinoma (though not present in all cases). If present, the diagnosis of adenosquamous carcinoma can be excluded [53]. Adenosquamous carcinoma has a worse prognosis than conventional SCC, presenting at an advanced stage, with frequent recurrences and dissemination despite surgical treatment with adjuvant chemoradiotherapy [54, 55].

*Lymphoepithelial carcinoma* is defined as a poorly differentiated SCC with prominent lymphoplasmacytic infiltration of the stroma. It resembles morphologically nonkeratinizing undifferentiated carcinoma of the nasopharynx. In contrast to nasopharyngeal SCC, it is only rarely EBV-positive but may harbor high-risk HPV [56–58]. It expresses pan-keratin and often squamous markers (p40, p63, CK5/6). It must be distinguished from melanoma and lymphoma by using proper immunohistochemistry, particularly when morphologic and immunohistochemical features of squamous differentiation are missing. The prognosis is similar to conventional SCC [59].

## Other Malignant Tumors

Other malignant tumors may arise in the hypopharynx, larynx, trachea and parapharyngeal space, e.g., adenocarcinomas, hematolymphoid neoplasms, melanoma, neuroendocrine and soft tissue tumors, as well as metastases, together accounting for up to 5% of malignant tumors at these locations. Laryngeal cartilaginous tumors are the only non-SCC malignancy of the larynx, hypopharynx, and trachea to be addressed in Chapter 3 of the new edition.

It is important to be aware of the significant progress and new insights into the genetic background of neoplasms, resulting in new tumor entities or tumour subtypes, e.g., in the field of salivary glands, hematolymphoid and soft tissue neoplasms. Furthermore, we are facing discoveries of new prognostic and predictive factors and the introduction of new targeted treatment modalities in various tumors.

It is also important to use the revised classification of neuroendocrine tumors of the head and neck, reflecting the unified WHO/IARC terminology [60]. The diagnosis of neuroendocrine carcinoma (NEC), for example, is now used only for poorly differentiated neuroendocrine tumors, further classified as either small cell or large cell NECs. Neuroendocrine tumors (NETs) are well differentiated tumors subclassified as NET grade 1, 2 or 3 on the basis of morphology, number of mitoses per 2 mm^2^ and the Ki67 proliferation index. Another potential diagnostic feature to distinguish NET grade 3 from NEC is lost expression of Rb and aberrant expression of p53 in NEC [61, 62].

When dealing with these tumors in the hypopharynx, larynx, trachea and parapharyngeal space, they must be diagnosed and classified according to criteria described in specific chapters.

## Cartilaginous Tumors

Chondrosarcoma (CS) is the most common laryngeal sarcoma, while its benign counterpart, chondroma is exceedingly rare [63, 64]. Both tumors arise in laryngeal hyaline cartilages, the most common site is the cricoid cartilage. New data of pathogenesis of central-type CS has been recently linked to isocitrate dehydrogenase 1 and 2 gene mutations in 12% of cases [65]. Chondroma and CS grow slowly as submucosal, lobulated endolaryngeal mass. The greatest dimension is an important parameter to differentiate both neoplasms: chondromas are usually less than 2 cm in diameter while CS can be as large as 12 cm, median 3.5 cm [66–69].

Microscopically, chondromas resemble mature hyaline cartilage with evenly distributed chondrocytes, while cellular pleomorphism, mitoses and multinucleated chondrocytes in a single lacunar space, and destructive invasion characterize CS. It is classified in terms of progressive atypias into three grades: the most frequent are low-grade (grade 1) and intermediate-grade (grade 2), while high-grade (grade 3) is very rare [67, 70,71]. The morphologic characteristics of conventional CS are well known, so the main stress has recently been devoted to three subtypes: clear cell CS is characterized by a sharp transition of the conventional type to a proliferation of large polygonal clear cells with distinct cellular membranes but without chondroid matrix. Most conventional and clear cell CSs behave as low-grade tumors¸ and have a prolonged clinical course with local recurrences and low risk of dissemination [72]. Dedifferentiated type CS has also been described in the larynx, but it is exceedingly rare. It is characterized by a biphasic pattern, with well-differentiated CS and a high-grade non-cartilaginous sarcoma. Morphologically, it can resemble undifferentiated sarcoma, osteosarcoma, rhabdomyosarcoma, leiomyosarcoma or angiosarcoma [73, 74]. Laryngeal mesenchymal chondrosarcoma is extremely rare, composed of small, round blue cells and islands of differentiated hyaline cartilage [75].

Differentiation between low-grade CS and chondroma can be extremely difficult, and is only possible after examination of the entire specimen. Chondromas are smaller than CS and less cellular, with less pleomorphism, lacking mitoses and necrosis. Recent study of 16 conventional CSs revealed that up to 60% of laryngeal CS arise in association with preexisting chondromas [69].

Function preserving surgery is the treatment of choice for both tumors. Prognosis is favorable. CS is characterized by a slowly progressive growth, with frequent recurrences, which are related to incomplete surgical excision and/or higher tumor grade [64, 67, 76].

## Squamous Papilloma and Papillomatosis

Squamous papillomas/papillomatosis (SP) are the most common benign epithelial tumors of the larynx, induced by low-risk HPV, primarily types 6 and 11. Single or multiple papillary tumors with frequent recurrences, affecting children and adults, and a propensity to spread to adjacent areas are recognized clinical characteristics. The first report of an internationally documenting decline of juvenile-onset SP incidence in children following a quadrivalent HPV vaccination program was published in 2018 [77]. Frequent recurrences of SP can be considered a consequence of the long-term persistence of a single viral genomic variant, rather than repeated reinfection with novel HPV strains [78]. The course of disease is unpredictable, ranging from mild disease and spontaneous remission to an aggressive disease with a requirement for frequent surgical procedures and eventual spread to adjacent regions with a protracted clinical course and potential life-threatening narrowing of the airways [79, 80]. Various studies have linked the unpredictable clinical course of SP also with host-specific genetic and immunological factors. Altered innate immunity could contribute to the anti-inflammatory immune response, causing the HPV 6/11 infection to persist in patients with SP [81–83].

The microscopic features of SP include irregularly scattered clusters of koilocytes in the upper half of the squamous epithelium, the only visible cytopathic effect of HPV infection. In situ hybridization can differentiate HPV status in infected cells: a diffuse nuclear staining pattern is consistent with episomal HPV DNA, while tiny punctate signals are related to the integrated form of HPV in the host cell chromosome [84, 85]. Dysplasia may be present, but there is no widely accepted specific criteria for dysplasia in SP. According to our experience, mild nuclear atypia and increased mitotic activity are often seen, probably reflecting viral replication. High-grade dysplasia is typically seen in recurrent SP, it has been described in up to 21.5% of patients [86]. Histological monitoring of all SP samples is thus required after each surgical procedure for the early detection of potentially risky morphological epithelial changes [87].

There is no single and definitive therapy for SP. The quadrivalent HPV vaccine has had a favorable influence on the speed of growth of SP, and prolonged intervals between surgical procedures in the majority of investigated patients [88]. Malignant transformation of SP into SCC is a rare event, reporting in 1–4% of cases of SP [85, 86, 89]. The role of dysplasia as a predictor of aggressive disease or risk of malignancy is controversial. Some authors suggest that histological changes are not a good predictor of potential malignant transformation [90], while others describe high-grade dysplasia in SP to be a risk factor for malignant transformation [86].

## Conclusions

SCC and its variants are by far the most common malignancies of the larynx, hypopharynx and trachea. The most important novelty is the introduction of new targeted treatment—checkpoint inhibitors, with a new task for pathologists, who may help to predict the response to treatment by analyzing the expression of targeted proteins in biopsy samples. Precancerous lesions remain a controversial topic; we are still searching for a reliable diagnostic marker to surpass the subjectivity in biopsy diagnosis, with a few potential new candidate markers on the horizon, e.g., stem cell markers. Other tumor types are relatively rare at these locations. They are dealt with in specific chapters.

## Data Availability

Data supporting the findings are available from the corresponding author [N.Z.], upon reasonable request.
